# Phenotypic and genetic analyses of claw lesions in TMR Holstein herds in South Africa

**DOI:** 10.5194/aab-69-1-2026

**Published:** 2026-01-07

**Authors:** Robyn C. Joubert, Bernice E. Mostert, Andries Masenge, Esté van Marle-Köster

**Affiliations:** 1 Department of Animal Science, University of Pretoria, Pretoria, 0028, South Africa; 2 Department of Statistics, University of Pretoria, Pretoria, 0028, South Africa

## Abstract

Claw lesions in dairy cattle pose a significant risk to dairy farmers worldwide in terms of animal welfare concerns and economic profitability. However, the use of different data sources, classification systems, and definitions of reference groups limits the comparison across herds and decreases the usability of the recordings for phenotypic and genetic analyses. In South Africa, information on claw lesions is not routinely collected by dairy farmers and data are limited to hoof trimmers recording lesions during preventative trimming or as needed by producers. Records of the most common claw lesions scored by a local hoof trimmer in five Holstein herds between January 2014 and December 2023 were used, including interdigital phlegmon (F), heel horn erosion (E), sole ulcers (SU), sole haemorrhage (SH), and two combined traits, one representing digital and interdigital dermatitis (DDID) and the other representing white line disease and white line separation (WLDS). The majority of lesions recorded were infectious (40.87 %), with DDID showing the highest incidence (39.58 %). Phenotypic associations may provide valuable information for hoof trimmers regarding the practical prevention, management, and treatment of lameness on-farm. A large, statistically significant odds ratio of 4.39 exists between DDID and E (95 % confidence interval: 3.55 to 5.43, P<0.0001 ). Within the non-infectious lesions, SH is moderately positively associated with total non-infectious lesions ( |ϕ|=0.43 , P<0.01 ) and the occurrences of SU and WLDS are both strongly positively associated with total non-infectious lesions ( |ϕ|=0.54 , P<0.01 and |ϕ|=0.58 , P<0.01 , respectively). In addition, the relationships observed among non-infectious lesions (SH, SU, and WLDS) are moderate to strong. The occurrence of DDID is moderately positively associated with the occurrence of infectious lesions in the front feet ( |ϕ|=0.39 , P<0.01 ) and strongly associated with the rear feet ( |ϕ|=0.89 , P<0.01 ). The occurrence of WLDS is moderately positively associated with the occurrence of total non-infectious lesions in the front feet ( |ϕ|=0.37 , P<0.01 ), with a stronger correlation with the occurrence of non-infectious lesions in the rear feet ( |ϕ|=0.46 , P<0.01 ). The occurrence of non-infectious lesions in the rear feet is moderately associated with the occurrence of SU ( |ϕ|=0.42 , P<0.01 ) but strongly associated with SH ( |ϕ|=0.57 , P<0.01 ). Heritability estimates ranged from 0.02 for DDID to 0.08 for the total lesions category (representing the presence or absence of at least one claw lesion on any foot). Phenotypic correlations and heritability estimates indicate that claw lesion data have the potential to be used for genetic evaluation of hoof health; therefore, the simplification and standardization of hoof lesion data collection should be encouraged.

## 1 Introduction

Claw lesions in dairy cattle have been widely reported as the primary cause of lameness (Huxley, 2013; Solano et al., 2016), which raises animal welfare concerns (Oehm et al., 2019; Sadiq et al., 2019) and affects the economic profitability of dairy farms due to reduced milk yield and fertility and an increased risk of premature culling (Chapinal et al., 2013; Afonso et al., 2020). Among lesions reported, digital dermatitis (DD), sole ulcers (SU), and white line (WL) lesions tend to be the most common in dairy herds worldwide (Christen et al., 2015; Solano et al. 2016; Shearer and Van Amstel, 2017), as well as in South Africa (Joubert et al., 2023).

Breeding strategies to improve functional traits such as fertility and udder health (Heringstad et al., 2012; Fleming et al., 2018) have proven to be successful, but the same progress has not been made in claw conformation and lesions (Egger-Danner et al., 2015). Selection for claw traits presents a more permanent solution to the problem, and researchers agree that claw-trimming data can be used in genetic evaluations for genetic selection (Koenig et al., 2005; Chapinal et al., 2013; Ødegård et al., 2013). However, the use of different data sources, classification systems, and definitions of reference groups limits the comparison across herds and decreases the usability of the recordings for phenotypic and genetic analyses (Charfeddine and Pérez-Cabal, 2017; Heringstad et al., 2018; Afonso et al., 2020). Despite guidelines by the International Lameness Committee (ILC) and the International Committee for Animal Recording (ICAR, 2020), different recording systems are used by hoof trimmers within and between countries (Oehm et al., 2019; Afonso et al., 2020). Different countries collect vastly different amounts of data on claw lesions, collecting information on between 6 and 20 different traits, with the level of information also varying widely, from cow-level information to single-claw information, and several countries also record information on the severity grades of the lesions observed (Christen et al., 2015). In addition to the difficulty and effort of performing routine claw trimming, datasets often lack complete pedigrees and tend to be limited in size for genetic evaluations (Heringstad et al., 2018). Accurate genetic analysis requires regular, consistent phenotypic recording, which means that it is important that the description and recording of claw lesions are made as easy as possible (Egger-Danner et al., 2015; Zavadilová et al., 2021; Joubert et al., 2023). In addition, specific phenotypic associations between individual claw lesions and categories may provide valuable information for hoof trimmers regarding the simplest and most practical manner to perform recording on-farm to aid in the practical prevention, management, and treatment of lameness (Joubert et al., 2023).

Phenotypic correlations between individual and combined claw lesion scores have been estimated by a number of researchers and tend to be highly variable due to a multitude of factors, including the recording methodology and completeness, as well as the person undertaking the recording (i.e. farmer versus veterinarian). Weak positive correlations were found by Van der Waaij et al. (2005) between sole haemorrhage (SH) and WL ( +0.10 ), WL and SU ( +0.09 ), and SH and SU ( +0.08 ), while Häggman and Juga (2013) found mostly weak negative correlations between the same lesions: -0.24 , -0.12 , and -0.18 , respectively. Stronger, positive phenotypic correlations of +0.18 between SH and both DD and heel horn erosion (E); +0.35 , +0.38 , and +0.42 between WL and E, DD, and SH, respectively; and +0.38 , and +0.51 between E and DD were reported by Capion et al. (2009). In addition, their combined trait analysis of double sole, interdigital hyperplasia, and sole ulcer showed positive correlations with both DD and E ( +0.14 and +0.26 ).

In genetic evaluations, claw disorders are generally defined as binary traits and analysed using linear animal models, which ignore repeated incidence, or threshold models, which take multiple occurrences into account (Malchiodi et al., 2017; Heringstad et al., 2018). Heritability estimates published for individual claw lesions tend to vary, with estimates from logistic and threshold models being slightly higher than those from linear animal models (Heringstad et al., 2018). In terms of the infectious lesions, DD (0.07–0.16) and interdigital hyperplasia, or IH (0.01–0.39), tend to have the highest heritability, and interdigital phlegmon, or F (0.01–0.06), tends to have the lowest, while SU generally has the highest estimated heritability under the non-infectious lesions (0.03–0.17), with the estimated heritability of SH tending to be the lowest, at between 0.02 and 0.09 (Van der Spek et al., 2013; Pérez-Cabal and Charfeddine, 2015; Malchiodi et al., 2017; Oliveira Junior et al., 2021). Genetic correlations between individual claw disorders tend to be low, ranging from -0.18 between DD and SU to +0.18 between SU and IH (Van der Waaij et al., 2005). Categorizing claw diseases into two groups (dermatitis and heel horn erosion versus sole haemorrhage and sole ulcer), Buch et al. (2011) found that genetic correlations between traits within the groups were high ( +0.87 and +0.73 , respectively), while genetic correlations between traits in different groups were low ( ≤+0.23 ). The low correlation ( +0.08 ) they found between infectious and non-infectious lesions was confirmed by Chapinal et al. (2013). Digital dermatitis has been shown to have positive correlation with all the other claw diseases, with the largest correlation with IH at +0.57 (Malchiodi et al., 2017).

Despite the low heritability of claw traits, researchers agree that there is sufficient genetic variability for genetic selection to be a viable option for improving these in dairy cattle (Van der Waaij et al., 2005; Van der Spek et al., 2013; Pérez-Cabal and Charfeddine, 2015). A number of European and Scandinavian countries, including the Netherlands, Denmark, Finland, Sweden, and Norway, have implemented routine genetic evaluation of claw health, and results indicate that, although heritabilities for claw disorders are generally low, it is possible to produce reliable breeding values using data currently available (Heringstad et al., 2018). In South Africa, information on claw lesions is not routinely collected by dairy farmers, and data are limited to hoof trimmers recording lesions during preventative trimming or as needed by producers. In addition, the number of dairy cows participating in milk recording in South Africa does not compare favourably with countries in the rest of the world. In 2018, only approximately 13 % of the national herd participated in official milk recording with either the Agricultural Research Council (ARC) or SA Stud Book (https://my.icar.org/stats/list, last access: 20 September 2024) (ICAR, 2024). This has resulted in limited pedigrees and smaller complete datasets being available for genetic analysis.

In this study, the aim was to analyse phenotypic and genetic associations for (a) total lesions (TL), (b) infectious lesions (IL), (c) non-infectious lesions (NL), and (d) digital and interdigital dermatitis (DD) in South African Holstein cattle managed under a total mixed ration (TMR) system.

## 2 Material and methods

Ethical approval for the study was granted by the University of Pretoria Ethics Committee in the Faculty of Natural and Agricultural Sciences (NAS292/2020). Five herds of Holstein cattle receiving a TMR and housed in dirt lots and/or free-stall housing systems were selected for this study, based on the availability of hoof-trimming data, pedigree information, and participation in the South African National Milk Recording Scheme. All five farms incorporated routine hoof trimming in their management protocol, either as part of their dry cow programme or simply as a lameness treatment tool using *The Claw Lesion Identification in Dairy Cattle* brochure, co-developed by Zinpro^®^ Corporation (D40-08-30-07; Eden Prairie, MN, USA) and the Zinpro^®^ Corporation (2024), as the reference for lesion identification. Two trimmers working at the same company are represented in this data, with Trimmer 1 having worked on four of the five farms and Trimmer 2 having worked on the remaining farm. Both trimmers were trained in the same methodology and quality control steps by the trimming company, and, because each trimmer was responsible for their own herds (i.e. no cross-over), it was assumed that any effect would be an included fixed effect of herd-year-season. Herd size data (Table 1) received from the SA Stud Book Association (SA Stud Book, Bloemfontein, South Africa) refer to female animals on the farm, including young heifers, first-calving heifers, dry cows, and all milking cows in the herds.

**Table 1:** Total herd size per farm per year over the 10-year study period.

Farm	2014	2015	2016	2017	2018	2019	2020	2021	2022	2023	Average
A	708	734	757	665	543	769	998	1046	1105	1088	841
B	1111	1380	1626	1735	1701	1985	2008	1959	1649	1715	1833
C	526	510	614	633	608	826	1012	979	1478	1052	824
D	472	365	793	813	770	1225	1306	1385	1378	1421	967
E	1509	1545	1628	1690	1724	2215	2149	–^∗^	–^∗^	–^∗^	1780

^∗^ Farm E stopped milk recording in 2021, so herd size data are not available for 2021, 2022, or 2023.

Claw lesions were recorded in these herds between January 2014 and December 2023. Trimming data were provided in hard copy and included general information such as the date of visit, farm and cow identification, and information relating to the identification of lesions by limb, foot, and claw. Data were electronically captured into Microsoft Excel worksheets (Microsoft Corporation, 2018) for quality control, data editing, and further analysis.

Records of the most common claw diseases, combined digital and interdigital dermatitis (DDID), interdigital phlegmon (F), heel horn erosion (E), sole ulcer (SU), sole haemorrhage (SH), and combined white line disease and white line separation (WLDS), scored by the hoof trimmer between 2014 and 2023, were used in the present study. Claw lesions were recorded per cow as the presence or absence of individual infectious lesions, DDID, F, and E or as the presence or absence of individual non-infectious lesions, including SU, SH, and WLDS. Three additional categories were also evaluated: total lesions (TL), total infectious lesions (IL), and total non-infectious lesions (NL), representing the presence of at least one of each category of lesion on any foot. The presence of individual total lesions in front and rear feet were also evaluated. Many cows were trimmed more than once within a lactation and also trimmed in more than one lactation. However, for this analysis, only the information from the first recorded trimming was used (Uggla et al., 2008; Laursen et al., 2009). The edited dataset included 10 236 cows with data from multiple trimmings, amounting to a total of 23 334 hoof-trimming records collected from five Holstein dairy herds between 2014 and 2023. Of these, 11 283 cows were identified as registered animals with identity numbers recorded at SA Stud Book.

### 2.1 Phenotypic analyses

The edited dataset used for phenotypic analyses consisted of 3650 unique cows with only their first trimming records per year included. Some cows had more than one disorder, leading to a mismatch in the total of prevalence records and the percentage of cows with a claw disorder. The non-parametric Spearman correlation was performed to test for correlations between the various lesions and lesion categories using the statistical software SAS^®^ 9.4 (SAS Institute Inc., 2018). The Spearman rho is equivalent to the ϕ coefficient used to measure the association between dichotomous data. The Spearman rho coefficient ranges between -1 and 1, with ϕ=1 implying a perfect positive association in which the presence of one variable perfectly predicts the presence of the other variable and with ϕ=-1 indicating that the presence on one variable perfectly predicts the absence of another variable (Table 2). In addition, the odds ratios were calculated to investigate the probability of specific lesions influencing the occurrence of each other.

**Table 2:** ϕ coefficient categories (Akoglu, 2018).

ϕ coefficient	Strength of association
|ϕ|<0.1	Very weak/negligible association
0.01≤|ϕ|<0.3	Weak association
0.03≤|ϕ|<0.5	Moderate association
0.5≤|ϕ|<0.7	Strong association
|ϕ|≥0.7	Very strong association

### 2.2 Genetic parameters

The final study population used to estimate genetic parameters amounted to 3650 unique animals with repeated trimming measurements (11 283 records). The pedigree file was generated by tracing the pedigrees of these cows three generations back, and the final file contained the relationships of 7921 animals.

Genetic parameters were estimated for claw lesions (TL, IL, NL, and DD) with restricted maximum likelihood (REML) using analytical gradients, fitting a multi-trait linear animal model using the VCE 6.0 software package (Groeneveld et al., 2008). The model (Eq. 1) included linear regression of age in months and the fixed effect of herd-year-season. Random effects included only the additive genetic effect (animal), as there were not enough repeated records to warrant including a permanent environmental effect.

1 $$y_{itklm}=\mu +{\text{hys}}_{tk}+{\text{age}}_{tl}+{\text{animal}}_{it}+e_{ijklm},$$

where $y_{itklm}=$ observation of trait t on animal i ( t= total, infectious, or non-infectious claw, or digital dermatitis); μ= overall mean; hys ${}_{tk}=$ fixed effect for trait t of the contemporary group k constructed by animals measured in the same herd, year, and season; age ${}_{tl}=$ linear regression modelling the effect of age at measurement on trait t ; animal ${}_{it}=$ random direct additive genetic effect of animal i ; and $e_{ijtklm}=$ random residual error.

## 3 Results

### 3.1 Phenotypic analyses

Across 3650 unique records, 56.32 % of cows in this study exhibited at least one claw lesion (whether infectious or non-infectious) (Table 3). The majority of lesions recorded were infectious (40.87 %) versus only 9.74 % non-infectious lesions. The highest incidence was for DDID, with almost 40 % of records indicating the occurrence of digital or interdigital dermatitis in at least one foot.

**Table 3:** Cow-level prevalence (number and percentage) of the claw disorders recorded on five TMR Holstein farms in South Africa between 2014 and 2023.

Claw disorder	No. lesions present	Lesion presence (%)
Total lesions	1315	56.32
Total infectious lesions	1059	40.87
Digital and interdigital dermatitis	1035	39.58
Heel erosion	410	12.65
Interdigital phlegmon	36	0.99
Total non-infectious lesions		9.74
Sole ulcer	102	2.87
Sole haemorrhage	63	1.76
White line disease and separation	111	3.14

Spearman correlation coefficients between the different lesions and categories of lesions presented in Table 4. Negligible negative correlations were observed between total individual infectious and non-infectious lesions ( P<0.05 ), while within the infectious lesions category, total DDID was weakly positively associated with total E ( |ϕ|=0.240 , P<0.01 ). Total E and total F were weakly positively associated with total infectious lesions ( |ϕ|=0.235 , P<0.01 and |ϕ|=0.156 , P<0.01 ), while DDID was almost perfectly associated with the occurrence of total infectious lesions (( |ϕ|=0.984 , P<0.01 ).

**Table 4:** Spearman correlation coefficients between total lesion categories. Bold font denotes correlations described in text.

	E (total)	F (total)	DDID (total)	IL (total)	SU (total)	SH (total)	WLDS (total)
E (total)							
F (total)	0.01						
DDID (total)	**0.24** ^a^	0.01					
IL (total)	**0.24** ^a^	**0.16** ^a^	**0.98** ^a^				
SU (total)	$-0.03^{b}$	-0.02	0.02	0.01			
SH (total)	$-0.03^{a}$	-0.01	$-0.07^{a}$	$-0.07^{a}$	0.02		
WLDS (total)	$-0.04^{a}$	-0.02	$-0.04^{a}$	$-0.04^{a}$	-0.02	0.00	
NL (total)	$-0.06^{a}$	-0.02	$-0.05^{a}$	$-0.06^{a}$	**0.54** ^a^	**0.43** ^a^	**0.57** ^a^

^a^ P<0.01 ; ^b^ P<0.05 . E: heel erosion; F: interdigital phlegmon; DDID: digital and interdigital dermatitis; IL: total infectious lesions; SU: sole ulcer; SH: sole haemorrhage; WLDS: white line disease and separation; NL: total non-infectious lesions.

Within the non-infectious lesions, SH was moderately positively associated with total non-infectious lesions ( |ϕ|=0.43 , P<0.01 ) and the occurrences of SU and WLDS were both strongly positively associated with total non-infectious lesions ( |ϕ|=0.54 , P<0.01 and |ϕ|=0.57 , P<0.01 , respectively). While the association between DDID and E within infectious lesions is weak, the relationships observed among non-infectious lesions (SH, SU, and WLDS) are moderate to strong.

In Table 5, the Spearman correlations indicate that E was weakly positively associated with the occurrence of DDID in both front and rear feet ( |ϕ|=0.11 , P<0.01 and |ϕ|=0.24 , P<0.01 , respectively). Furthermore, the occurrence of DDID was moderately positively associated with the occurrence of infectious lesions in the front feet ( |ϕ|=0.39 , P<0.01 ) and strongly associated with the rear feet ( |ϕ|=0.89 , P<0.01 ). The occurrence of F was weakly positively correlated with the occurrence of infectious lesions in both the front and rear feet ( |ϕ|=0.13 , P<0.01 and |ϕ|=0.16 , P<0.01 , respectively).

**Table 5:** Spearman correlation coefficients between individual lesions in front and rear feet and totals on lesion categories. Bold font denotes correlations described in text.

	E	F (front)	F (rear)	DDID (front)	IL front (total)	IL rear (total)	NL front (total)	NL rear (total)
E								
F (front)	0.06^a^							
F (rear)	-0.03	-0.01						
DDID (front)	**0.11** ^a^	-0.01	0.01					
DDID (rear)	**0.24** ^a^	0.07^a^	$-0.04^{b}$	0.04^b^				
F (total)					**0.13** ^a^	**0.16** ^a^		
DDID (total)					**0.39** ^a^	**0.89** ^a^		
SU (total)							0.02	**0.57** ^a^
SH (total)							0.09^a^	**0.42** ^a^
WLDS (total)							**0.37** ^a^	**0.46** ^a^

^a^ P<0.01 ; ^b^ P<0.05 . E: heel erosion; F: interdigital phlegmon; DDID: digital and interdigital dermatitis; IL: total infectious lesions; SU: sole ulcer; SH: sole haemorrhage; WLDS: white line disease and separation; NL: total non-infectious lesions.

The occurrence of WLDS was moderately positively associated with the occurrence of total non-infectious lesions in the front feet ( |ϕ|=0.37 , P<0.01 ), with a stronger correlation with the occurrence of non-infectious lesions in the rear feet ( |ϕ|=0.46 , P<0.01 ). The occurrence of SU was moderately associated with the occurrence of non-infectious lesions in the rear feet ( |ϕ|=0.41 , P<0.01 ), while SH was strongly associated with the occurrence of non-infectious lesions in the rear feet ( |ϕ|=0.57 , P<0.01 ).

### 3.2 Genetic parameters

Heritability estimates were estimated for (a) total lesions (TL), (b) infectious lesions (IL), (c) non-infectious lesions (NL), and (d) digital and interdigital dermatitis (DDID), but only traits with a significant value are reported in Table 6.

**Table 6:** Heritability estimates and standard errors (SE) for lesion categories based on multi-trait linear animal modelling using VCE 6.0 ( n=3650 ).

Lesion category	$h^{2}$	SE
Total lesions	0.01	0.01
Total non-infectious lesions	0.05	0.04
Digital and interdigital dermatitis	0.02	0.02

## 4 Discussion

This study aimed to investigate phenotypic and genetic associations between individual claw lesions and categories of claw lesions in five South African Holstein herds fed a TMR in order to gain a deeper understanding into the occurrence of lesions and make recommendations for future recording of claw lesions in South Africa.

Mhlongo (2019) published the first article on the prevalence of claw lesions in South Africa, based on hoof-trimming data from 10 TMR herds in the same region as the current study. Of these 10, only the 5 herds included in the current study offered access to phenotypic and genetic data through their registration with SA Stud Book and the South African Holstein Society. In addition, unfortunately, Farm E deregistered from the South African National Milk Recording Scheme in 2021. The five herds represented in this study are representative of four provinces in the central region of South Africa (Gauteng, Mpumalanga, the Free State, and Limpopo), and, while they only represent a small percentage of Holstein herds in the country that are managed under a TMR system, they were chosen based on the availability of both phenotypic and genetic data, as well as participation in the South African National Milk Recording Scheme. Unfortunately, there are very few herds in South Africa that qualify under these parameters, and the number is continually decreasing. The fragmentation of milk recording and the closure of the country's only local bull breeding station, together with the advent of genomic selection elsewhere in the world, has resulted in the local dairy industry being increasingly less interested in participating in national phenotypic recording.

South Africa has the second-largest average herd size in the world, second only to Saudi Arabia (Lacto Data, Milk SA, 2023). As is the case in the rest of the world, dairy farmers are trying to optimize economies of scale in order to remain profitable, but South African dairy farmers are more prone to retaining more replacement heifers than purchasing heifers from elsewhere (Meissner et al., 2013).

Over 50 % of cows in this study had at least one claw lesion, which is in agreement with previous researchers reporting a high prevalence of between 40 % and 70 % of cows having at least one type of hoof lesion (Manske et al., 2002; Van der Waaij et al., 2005; Sogstad et al., 2005; Buch et al., 2011; Chapinal et al., 2013). Studies by Van der Spek et al. (2013) and Croué et al. (2017) reported a prevalence of 55 % and up to 80 % incidence of at least one claw lesion being present in dairy cows in France. Infectious lesions were found to be 5 times more prevalent than non-infectious lesions in this study, with DDID being the most prevalent lesion overall and with WLDS, SU, and SH being the most prevalent non-infectious lesions. Similar findings were reported in previous studies (Malchiodi et al., 2017; Van Huyssteen et al., 2020). Despite increased awareness, repeated findings of high prevalence of claw lesions indicate that adoption of prevention and control strategies by producers remains low (Heringstad et al., 2018; Van Huyssteen et al., 2020). This may be due to underestimation of the problem, ambiguity regarding different scoring and reporting systems, or information overload on the part of the producer when too many individual lesions are reported on (or a combination hereof).

Phenotypic correlations between individual and combined claw lesion scores have been estimated by a number of researchers and tend to be highly variable due to differences in study population size, lesion scoring methodology (i.e. present versus absent or ordinal scoring according to severity), and lesion categorization (Van der Waaij et al., 2005; Capion et al., 2009; Häggman and Juga, 2013). The study of Van der Waaij et al. (2005) categorized interdigital dermatitis with heel horn erosion (IDHE) as a combined trait and evaluated digital dermatitis and interdigital hyperplasia as individual traits, while both Capion et al. (2009) and Häggman and Juga (2013) evaluated digital dermatitis and interdigital dermatitis separately and Häggman and Juga (2013) did not evaluate interdigital hyperplasia at all. In addition, while both Van der Waaij et al. (2005) and Häggman and Juga (2013) scored lesions as present or absent, Van der Waaij et al. (2005) only reported on lesions occurring in the rear legs and Capion et al. (2009) scored lesion severity on two different scales for different lesions. This emphasizes the complexity of comparing research results across studies.

The literature is divided regarding the correlation between sole ulcer and sole haemorrhage: Van der Waaij et al. (2005) found a weak positive correlation ( +0.08 ), and Häggman and Juga (2013) found a weak negative correlation ( -0.18 ), while Barden (2022) estimated a strong positive genetic correlation between sole haemorrhage and sole ulcer. However, by definition, SU is a continuous break in the epidermis of the sole horn that exposes the corium, of which SH is regarded as an early sign (Van der Waaij et al., 2005; Van Amstel and Shearer, 2006; Solano et al., 2016), so the correlation is expected to be higher than is indicated in this study.

Due to the small sample size in this study, only total lesion categories were evaluated, together with DDID due to its high prevalence. The combined trait of digital and interdigital dermatitis was very strongly associated with the occurrence of total infectious lesions, which probably reflects the prevalence of the infectious lesion recorded; DDID and E, along with E and F, were also weakly positively correlated. While the phenotypic association between individual infectious lesions is weak, the relationships observed among non-infectious lesions (SH, SU, and WLDS) are moderate to strong, suggesting that the non-infectious lesions are more closely related to each other than the infectious lesions are to one another. However, a large, statistically significant odds ratio of 4.39 was found between DDID and E (95 % confidence interval: 3.55 to 5.43, P<0.0001 ), indicating that the presence of one of these infections is associated with approximately a 4-fold increase in the odds of the other occurring. In the literature, claw disorders with low frequencies and similar biological causes are sometimes grouped together as an option to increase the number of records for analysis (Heringstad et al., 2018). Researchers have differed in their categorization over the years: Buch et al. (2011) grouped hygiene-related lesions (dermatitis and heel horn erosion) and feed- and housing-related lesions (sole ulcer and sole haemorrhage) together due to high genetic correlations within groups and low genetic correlations between groups; Johansson et al. (2011) added an additional category called malformation (CC) traits; Ødegård et al. (2013) suggested adding white line disease to the feed-related category and relabelling it laminitis-related lesions and then grouping infectious lesions together (E, DD, and F); Chapinal et al. (2013) also defined three categories as infectious, horn, and other lesions; and Dhakal et al. (2015) suggested simply categorizing groups as either infectious or non-infectious. Given the distinct management actions required for intervention regarding infectious versus non-infectious lesions, the categorization of Dhakal et al. (2015) frequently seems to be the simplest and most relevant for practical reasons.

Claw data are usually recorded as binary or categorical traits, and, in theory, threshold models are the most suitable to analyse these kinds of response variables (Chapinal et al., 2013; Pérez-Cabal and Charfeddine, 2015; Malchiodi et al., 2017). Heringstad et al. (2018) reported that heritability estimates from threshold models tended to be higher compared to those from linear models, ranging from 0.05 to 0.20 (Van der Waaij et al., 2005; Swalve et al., 2008; Malchiodi et al., 2017). However, linear models are easier to implement for the scale and scope of a routine genetic evaluation (Malchiodi et al., 2017), and previous research has not indicated relevant differences in results from the two models (Weller et al., 1988; Van der Waaij et al., 2005; Malchiodi et al., 2017).

The low heritability of combined digital and interdigital dermatitis (0.01) reported for this study is similar to that found by Pérez-Cabal and Charfeddine (2015) at 0.02 (0.004) using linear modelling, although they found a higher heritability using a threshold model (0.14). Previous researchers found a higher heritability for the combined digital and interdigital dermatitis trait than the present study: 0.07 using logistic modelling (Koenig et al., 2005) and 0.04 using linear modelling (Van der Spek et al., 2013). Van der Waaij et al. (2005) categorized two traits, interdigital dermatitis and heel horn erosion (IDHE) and DD, separately and found heritabilities of 0.05 and 0.10, respectively. Other researchers that categorized digital and interdigital dermatitis as two separate traits found a similar estimated heritability for DD (0.08 and 0.07, respectively), while the estimated heritability of interdigital dermatitis was not similar in these two studies: 0.09 versus 0.01, respectively (Swalve et al., 2008; Malchiodi et al., 2017). Similarly, the Canadian Dairy Network has published heritability estimates of 0.08 for DD and 0.05 for interdigital dermatitis (Butty et al., 2021).

The heritability of the total lesions category (representing the presence or absence of at least one claw lesion on any foot) was found to be 0.01. In their respective studies, Van der Spek et al. (2013) and Pérez-Cabal and Charfeddine (2015) also included a combined claw disorder trait and found the heritability to be 0.05. Chapinal et al. (2013) performed a similar study to the current experiment using a linear animal model and found an estimated heritability for any lesion as $h^{2}=0.08$ (0.01). The higher heritability estimates found in the literature are probably a reflection of the smaller sample size of this study.

The estimated heritability of total non-infectious lesions in the current study is 0.05, comparing favourably with previous research. Combining laminitis, interdigital hyperplasia, and white line disease records into a category called non-purulent claw disorders, Gernand et al. (2012) reported an estimated heritability of 0.07 using threshold modelling. The category of horn lesions (SH, SU, and WL) as defined by Chapinal et al. (2013) has a heritability of 0.02, and Dhakal et al. (2015) reported a heritability of 0.08 for total non-infectious lesions.

While genetic correlations were not investigated in this study, estimated correlations in the literature tend to vary widely (Johansson et al., 2011; Van der Spek et al., 2013), with a trend towards lower correlations between groups of traits (infectious versus non-infectious) than within groups, underlining the existence of two genetically distinct groups of claw lesions (Croué et al., 2017).

It has been over a decade since genomic prediction strategies have been implemented in the genetic evaluation of dairy cattle on a routine basis for over a decade, facilitated by genome-wide association studies (GWASs) that have identified significant variants related to economically important traits (Gutierrez-Reinoso et al., 2021; Sahana et al., 2023). Genomic information offers a great opportunity to develop a more comprehensive understanding of the genetic mechanisms involved in difficult-to-measure traits and those with low heritability, and more recent studies have started including health and welfare traits in such analyses (Hossein-Zadeh, 2024; Krupová et al., 2024). A number of researchers have attempted to investigate claw lesion traits using a genomic approach, although, as is the case in defining and recording claw lesions, here, too, trait definition and the ways of estimating the accuracy of genomic evaluation approaches vary widely among authors (Croué et al., 2019; Lai et al., 2020; Joubert, 2025).

Regular claw-trimming can provide valuable data regarding claw lesions in individual cows and can also provide an important insight into the health status of an entire herd or population. However, diverse documentation practices complicate the routine collection and use of such data (Christen et al., 2015). It has been shown that both specific claw lesions and categories of lesions are heritable and can be improved by selection. However, this requires regular, consistent phenotypic recording, so it is vital that the description and recording of claw lesions are made as easy as possible (Zavadilová et al., 2021; Joubert et al., 2023). Claw-trimming records can be used to improve our understanding of lesion occurrence and prevalence in (South African Holstein) herds in order to develop procedures that accommodate the most common circumstances in the field. Using data relating to phenotypic correlations between specific lesions and grouping lesions into categories based on their aetiology and management interventions, trimmers can simplify their recording sheets to encourage increased producer participation.

## 5 Conclusion

The prevalence of claw lesions in South African Holstein cattle fed a TMR is comparable to several studies from dairy cows elsewhere in the world. Similar issues are reported regarding accurate, consistent, and comparable phenotypic recording of lesion data by hoof trimmers. The phenotypic correlations and heritability estimates found in this and other studies indicate that incorporating some measure of claw health into breeding programmes has merit, but it is important that combined traits be used instead of individual lesions in order to maximize the amount of phenotypic data available. In addition, in order to make a real difference on the farm, producers need to be empowered with only the most relevant information to enable practical management interventions. Hoof lesion data collected by hoof trimmers have the potential to be used for genetic evaluation of hoof health; therefore, simplification and standardization of hoof lesion data collection should be encouraged.

## Data Availability

The datasets generated and/or analysed during the current study are not publicly available due to post-graduate research still being in process but are available from the corresponding author on reasonable request.
